# Commercial use of evidence in public health policy: a critical assessment of food industry submissions to global-level consultations on non-communicable disease prevention

**DOI:** 10.1136/bmjgh-2021-006176

**Published:** 2021-08-22

**Authors:** Kathrin Lauber, Darragh McGee, Anna B Gilmore

**Affiliations:** Department for Health, University of Bath, Bath, Somerset, UK

**Keywords:** health policy, public health, prevention strategies, nutrition

## Abstract

**Background:**

Ultra-processed food industry (UPFI) actors have consistently opposed statutory regulation in health policy debates, including at the WHO. They do so most commonly with claims that regulatory policies do not work, will have negative consequences or that alternatives such as self-regulation work well or better. Underlying this are often assertions that industry is aligned with principles of evidence-based policymaking. In this study, we interrogate if this holds true by exploring the extent and quality of the evidence UPFI respondents employed to support claims around regulatory policy, and how they did this.

**Methods:**

First, we identified all submissions from organisations who overtly represent UPFI companies to consultations held by the WHO on non-communicable disease policy between 2016 and 2018. Second, we extracted all relevant factual claims made in these submissions and noted if any evidence was referenced in support. Third, we assessed the quality of evidence using independence from UPFI, nature, and publication route as indicators. Lastly, where peer-reviewed research was cited, we examined if the claims made could be justified by the source cited.

**Results:**

Across 26 included consultation responses, factual claims around regulation were made in 18, although only 10 referenced any evidence at all. Of all 114 claims made, 39 pieces of identifiable evidence were cited in support of 56 claims. Of the 39 distinct pieces of evidence, two-thirds were industry-funded or industry-linked, with only 16 externally peer-reviewed. Over half of industry-funded or industry-linked academic articles failed to declare a conflict of interest (COI). Overall, of only six claims which drew on peer-reviewed *and* independent research, none appropriately represented the source.

**Discussion:**

UPFI respondents made far-reaching claims which were rarely supported by high-quality, independent evidence. This indicates that there may be few, if any, benefits from consulting actors with such a clear COI.

**Key questions**

**What is already known?**

- There is growing evidence that regulatory policies are more effective than voluntary industry measures in addressing obesity and dietary non-communicable diseases which pose a growing threat to public health.
- Ultra-processed food industry (UPFI) actors claim to be aligned with evidence-based policymaking, but nonetheless consistently oppose evidence-based regulation in favour of voluntary approaches.

**What are the new findings?**

- Our research shows that, in response to WHO consultations, UPFI groups made many factual claims to oppose regulatory policies and promote alternative measures, but only cited evidence in support of just over half of all instances.
- Most respondents did not make extensive use of evidence.
- The majority of the evidence cited to support factual claims lacked key indicators of quality such as independence or external peer review.
- Where industry respondents cited peer-reviewed research evidence, they often failed to represent the source accurately.

**Key questions**

**What do the new findings imply?**

- In line with previous research on commercial use of evidence, our study suggests that UPFI actors not only tend to provide evidence which lacks key quality indicators but also employ evidential practices which serve to create doubt about the public health evidence.
- As such, industry actors might not only feed lower-quality evidence into policy processes; mirroring discourse around science and evidence could also potentially bolster their credibility and that of their arguments.
- Thus, those developing public health policies or policy recommendations should reconsider if and how they engage with commercial actors.

## Introduction

Approximately 10 million global deaths per year are attributable to unhealthy diets,^1 2^ a key risk factor for non-communicable diseases (NCDs) such as cancers, cardiovascular disease, and type 2 diabetes.^3^ Over recent decades, it has become increasingly evident that industry self-regulation is less effective to improve diets than government regulation.^4–16^ Yet, and despite sustained calls by the public health community for comprehensive regulatory frameworks to safeguard children’s right to health in particular,^17–20^ such policies remain sporadic. This disconnect reflects the significant role played by politics, values, ideas and discourse, as well as the notion that all evidence is socially constructed,^21^ contestable and open to interpretation.^22–24^ Narrow conceptions of evidence-based policymaking (EBPM) largely fail to account for this real-world context in which evidence and policy are created.^25^ The alternative term evidence-*informed* policymaking has emerged more recently, explicitly acknowledging that while the best available evidence should be used, decisions are not purely based on technical considerations.^26^ Regardless of differences in terminology, it is clear that efforts to improve the uptake of knowledge in public health policy over recent decades have firmly positioned evidence as a source of power and legitimacy in decision-making.^25 27^ Crucially, this goes beyond the instrumental power of evidence and its producers or users, extending to the discursive power actors can derive from claims to scientific knowledge and authority.

The fundamentally political nature of policymaking is particularly noticeable where powerful commercial entities find themselves facing a threat of regulation: multinational companies involved in the sale of ultra-processed food and beverage products as well as their representative groups (the ultra-processed food industry, UPFI) have consistently engaged in ‘corporate political activity’ to prevent, delay, or weaken regulatory policies,^28–34^ which has been identified as a key barrier to effective dietary public health policy.^35 36^ Large volumes of research on the tobacco industry^37–40^ and now increasingly on the UPFI^30 32 41–45^ and other unhealthy commodity industries^46–49^ show that corporate actors’ ability to shape and use evidence in their own interest plays a key part in their policy-influencing strategies.^50^

Even preceding an acute policy debate, corporate influence on science can shape the body of evidence on a topic, thereby influencing what is perceived as a problem and which solutions are considered. Research funded by UPFI entities or conducted by academics with a conflict of interest (COI) appears more likely to reach conclusions that are favourable or simply non-threathening to the donor industry.^51–55^ Systematic reviews where authors declared a COI, for instance, were found to be five times more likely to conclude no positive association between sugar-sweetened beverage (SSB) consumption and weight gain than independent reviews.^53^

On the other hand, a less studied facet of the interface between public health policy, evidence, and corporations is the strategic use of evidence within the policymaking process. Existing public health research on this topic has focused predominantly on tobacco control^56–58^ and alcohol policy,^59–61^ with only two articles, to our knowledge, systematically exploring use of evidence within dietary NCD policy.^28 62^ Largely divisible into two analytical strands, examinations of the *nature* of evidence used by commercial actors and of *how* this evidence or, more broadly, the concept of evidence are used. The available research suggests that unhealthy commodity industry actors predominantly use evidence that is not independent and externally peer-reviewed—thus lower in quality—and where they do use scientific evidence, tend to misrepresent the source.^28 56–58 60 62^

This paper aims to combine both of these analytical strands to explore how UPFI actors promote their NCD policy preferences at the WHO. It builds on a previous study where we document how UPFI associations opposed regulatory approaches such as marketing restrictions, mandatory front-of-pack labelling, and particularly SSB taxation in consultations held to inform WHO recommendations.^63^ At their core, claims focused on conveying the narrative that regulatory policies would not have the desired public health effect, would lead to unintended negative consequences, and that alternatives to regulation would be equally or more effective. In line with earlier research,^30 43 64^ we showed that UPFI actors widely espoused the concept of EBPM and made prominent use of terms related to science and evidence to justify opposition to regulatory approaches. In light of these industry claims to take an evidence-based approach, we aim to investigate whether and how evidence was used to support factual claims about regulation in recent WHO consultations. Specifically, we ask:

1. To what extent did UPFI actors refer to evidence when making factual claims about policies?
2. What types of evidence did UPFI actors refer to when making factual claims about policies? Was it independent and peer-reviewed?
3. Where peer-reviewed research was cited to support factual claims, does the claim accurately reflect the source content?

To address the last research question, we draw on concepts from agnotology, a term coined by Proctor^65^ to describe the study of the deliberate spread of ignorance, which posits that policymaking may be shaped by so-called agnogenic practices, ‘methods of representing, communicating, and producing scientific research and evidence which work to create ignorance or doubt irrespective of the strength of the underlying evidence’.^62^ Previous research exploring agnogenic practices in consultations for UK tobacco plain packaging^58^ and the South African SSB tax^62^ found that corporate actors used techniques such as quoting evidence in misleading ways, mimicking scientific critique to contest the public health evidence supporting regulation, and excluding relevant evidence while promoting alternative narratives.

## Methods

To explore how evidence has been used by commercial actors in global-level policy spaces, we analysed UPFI responses to WHO consultations on NCD policy. Specifically, we focused on arguments against the statutory regulation of unhealthy foods and non-alcoholic beverages, assessing the evidence cited in this context for markers of quality. To establish whether peer-reviewed evidence was represented accurately, we also conducted a verification-oriented cross-documentary analysis which compares claims made with the cited source documents.^58 62^

### Data

We systematically searched the WHO Headquarters website for consultations held between 2016 and 2018 which covered dietary NCD policy, were global in scope, and for which all responses were published in full. Four consultations met our criteria: the *web-based consultation of the WHO Independent High-level Commission on NCDs*,^66^ the *consultation on the Member State-led draft outcome document for the WHO Global Conference on NCDs* (‘Montevideo roadmap’),^67^ and the consultations on *updating Appendix 3 of the WHO Global NCD Action Plan 2013–2020*^68^ and the *zero draft Shanghai Declaration on Health Promotion*.^69^ The consultations are described in more detail elsewhere.^63^ From the 393 total responses to these consultations, we extracted all responses made on behalf of the UPFI, starting with all private sector submissions (as categorised by WHO) to identify submissions overtly representing the UPFI (ie, corporations manufacturing ultra-processed foods/soft drinks or holding a financial interest in their sale, or business associations who self-describe as representing the latter). Of the identified 33 responses from UPFI actors—all business associations—we excluded six which were not in English^70–75^ and one which contained only a copy of the consultation document,^76^ leaving 26 submissions.

### Identification of factual claims and evidence used to support them

Our analysis concentrated on statements which opposed regulatory approaches to dietary NCDs, as we could not identify any which supported the introduction of new statutory regulation. Using Atlas.ti^77^ software, the lead author coded all instances within the 26 included submissions where factual claims—defined as statements which appear to convey a fact rather than a belief, opinion, or idea—were made in relation to policy effects. Thus, statements which merely referred to the existence of policies or commitments without discussing their effects were not included. Factual claims were coded into three core categories and two subcategories which we developed after in-depth reading of the documents (table 1). Where a sentence made more than one of the assertions below, these were counted as two separate claims.

**Table 1:** Categorisation framework for factual claims

Factual claim category	Detail
1: Regulation does not work	Claims that statutory approaches to regulating unhealthy products, in particular SSB taxes, do not have the intended benefits for public health, arguing that a policy will fail or has previously failed to reduce consumption of the target products.
1.1: The rationale for regulation is flawed	Claims which do not directly refer to policy effects, but question the causal mechanisms underlying obesity and dietary NCDs which regulatory approaches seek to tackle, for instance, the link between obesity and/or NCDs and the target products.
2: Regulation will have unintended negative consequences	Some respondents went further to suggest that regulatory policies may have negative economic consequences or will even be counterproductive, for instance, increasing the consumption of other unhealthy products.
3: Alternatives to regulation work well/better	Claims that alternatives to regulation—information campaigns, self-regulation or co-regulation—would work equally well or better than regulation to address obesity or dietary NCDs. This forms an important pillar of a broader argument that regulatory policies are not needed.
3.1: Compliance with self-regulation or co-regulation is high	Statements suggesting that industry compliance with self-regulation or co-regulation is high, thus implying positive effects without directly referring to public health outcomes.

NCDs, non-communicable diseases; SSB, sugar-sweetened beverage.

Next, we coded whether any evidence was referenced in support of the claim, and extracted it into a spreadsheet. We adopted a broad definition of evidence as formal and informal written sources, such as reports, journal articles, press coverage, blogs, and opinion pieces. We included all instances where evidence was formally cited (at the end of a page or submission), or referred to in the text, provided enough information was available to identify it through a web search. Links to general websites were not included as they do not clearly refer to a distinct piece of evidence. Where coding decisions were challenging or uncertain, this was resolved in discussion between the first and second author.

### Analysis

We conducted two separate analyses. First, we assessed the quality of all evidence referenced in support of factual claims on policy effects. Second, we assessed how peer-reviewed research was used in this context.

#### Analysis of evidential quality

We adopted the criteria from Evans-Reeves *et al*^78^ and Hatchard *et al*^56^ to assess the quality of evidence referenced: independence from the UPFI, nature of evidence and publication route (see table 2 for detail). *Independence* was assessed by first searching if the consultation submission itself stated a link between an UPFI entity and the evidence cited. If this was not the case, we went on to screen the cited piece of evidence for a funding or conflict or interest statement. If none was declared, we conducted web searches for the authors in combination with the name of the organisation which cited the evidence in its consultation response, and the four largest packaged food and soft drink companies (The Coca-Cola Company, PepsiCo, Nestlé, Mondelez).^79 80^ This sample was selected because large corporations have been found to be more involved in funding nutrition research compared with smaller companies and trade associations.^55^ We also read author curriculum vitaes and short biographies where available. Evidence was classified as clearly independent if it was published by an intergovernmental organisation or government, or if clear funding or COI statements were available and did not list any recent (<6 years) UPFI financial links, and web searches did not reveal any UPFI connections. The *nature of evidence* was categorised as research, opinion, strategy documents or raw data. To assess the origins of evidence, we categorised the *publication route* as either peer-reviewed journals and other academic outlets, intergovernmental organisations and governments, or publications by private companies and organisations. We separately assessed if academic sources had been externally peer-reviewed. For industry-funded and industry-linked academic publications, we also noted whether the source declared a COI. We ran descriptive analyses in IBM SPSS version 26.^81^

**Table 2:** Coding framework for cited evidence

Category	Codes	Description
Independence	Industry-funded	Statement included that the research was financially supported by a food industry entity (ie, UPFI corporations, business associations, and other organisations majority funded or run by UPFI corporations).
	Industry-linked	No statement or other indication that the research was directly funded by the UPFI, but evidence of other connection: for example, author(s) or publishing organisation has financial links to UPFI entities (within five years of publication).
	Appears independent	Insufficient or no information provided on funding and COI on document/organisational website, but no evidence of prior connection with the UPFI can be found through additional searches.
	Clearly independent	The source is published by a government or multilateral body or (if published by an academic journal or private organisation) provides detailed information about the funding of the work or clearly states independence from the food industry. Searches for author(s) do not show any evidence of links to industry (within 5 years of publication).
Nature of evidence	Research	Primary or secondary research, including, for example, surveys, experimental studies, literature reviews, and qualitative studies.
	Opinion	Written pieces largely based on opinion, including those with some supporting evidence. This may, for instance, include blogs, commentaries, and editorials.
	Strategy documents	Outlines a strategy or plan of action, for example, organisation annual report including evaluation of previous year and plans for the future. This may include a combination of research, opinion and statistics/data.
	Raw data	Data without the underlying methodological detail or interpretation found in research studies.
Publication route	Peer-reviewed journals and other academic outlets	Published by peer-reviewed journals or other academic outlets such as university websites.
	Official government or international organisation publications	Publications by national government bodies or international organisations such as the United Nations and its agencies.
	Publication by private companies and organisations	Publications by private companies, consultancies, think tanks or other organisations (includes private international organisations such as the World Economic Forum).
	Published by the press	Print or online news publication.
External peer review	Peer-reviewed	Published in a peer-reviewed journal (exceptions, checked on a per-case basis: conference abstracts, commentary pieces).
	Not peer-reviewed	Includes, for example, news articles, blogs, company reports, private research reports and research commissioned by government/multilateral bodies.

COI, conflict of interest; UN, United Nations; UPFI, ultra-processed food industry.

#### Analysis of use of scientific evidence

To examine how scientific evidence was used by industry actors, a verification-oriented cross-documentary analysis was conducted for all instances where peer-reviewed research articles were cited to support relevant factual claims. In each case, we compared the statement made with the cited source to assess whether the claim reflected the latter. During this process, we noted where agnogenic practices occurred, drawing on the typology developed by Ulucanlar *et al*^58^ based on an analysis of tobacco industry misuse of scientific evidence (table 3).

**Table 3:** Analytical framework for use of scientific evidence, adapted from Ulucanlar *et al*’s evidential strategies^58^

Industry practice	Description
Misleading quoting of evidence	Inaccurate reporting from published scientific research, including misquoting, selective quoting or misinterpretation.
Mimicked scientific critique	Detailed inspection of published research, superficially resembling scientific peer review and using scientific terminology. For instance, seeking methodological perfection or insisting on methodological uniformity.
Evidential landscaping	The promotion of alternative evidence or exclusion of relevant public health evidence.

## Results

### Factual claims and evidence used to support them

**Figure 1:**
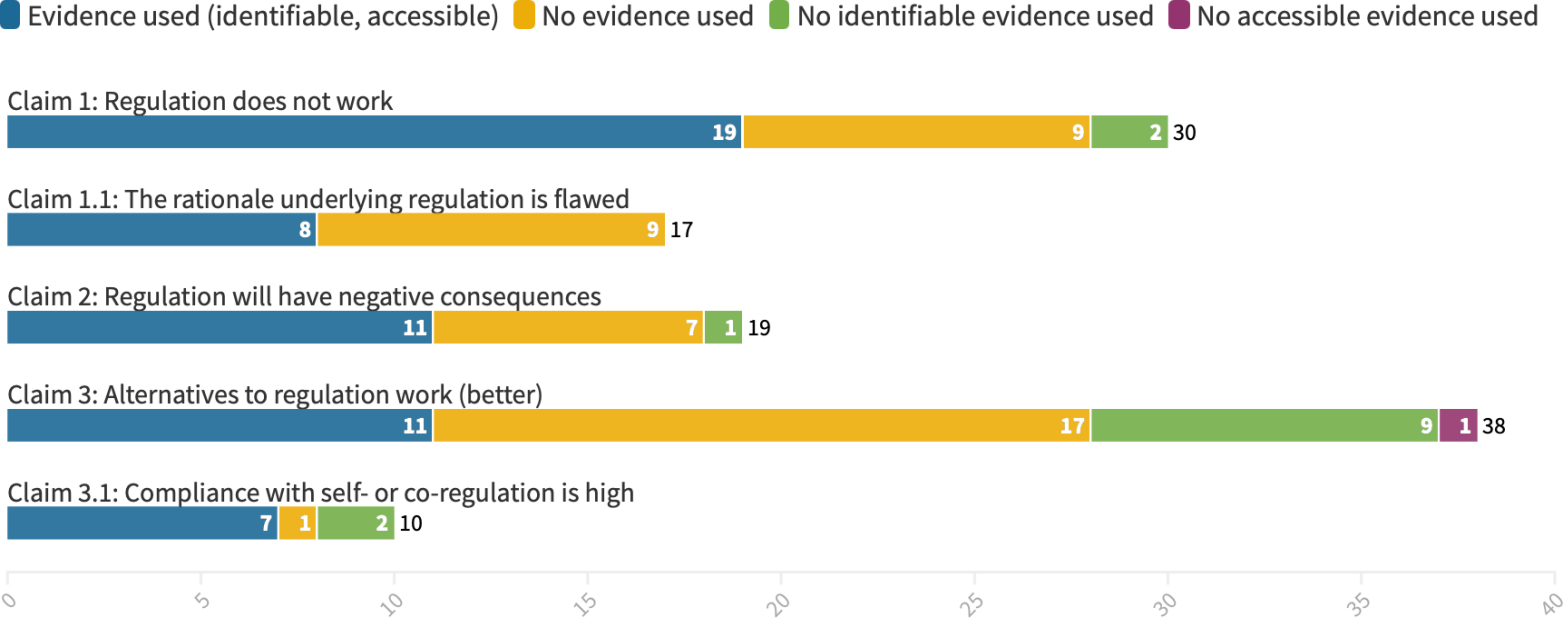
Evidence used to support different types of claims. Created using *flourish studio*.^81^

UPFI actors made 114 separate factual claims in 18 of the 26 included submissions (figure 1). Of these 114 claims, 66 challenged regulatory policies (claim categories 1, 1.1, 2) and 48 supported alternative policies such as self-regulation or co-regulation (claim categories 3, 3.1). With the exception of two claims related to advertising, all claims countering regulatory policies focused on fiscal policy. The promotion of alternatives to regulation spanned across a wide range of measures such as voluntary reformulation and labelling, advertising codes and public–private partnerships.

Only 71 of the 114 claims in 10 of the 26 responses actually referred to any evidence. Yet, only in 56 of the 71 factual claims citing evidence was this evidence identifiable: the remaining lacked key information such as title, year published, or author, and one item was paywalled market research data.

The remaining 43 factual claims were made without any reference to a specific piece of evidence, despite some making strong statements with casual references to ‘the evidence’, as exemplified by the Italian business association Federalimentare^82^ which asserted that

[a]vailable scientific evidence on sugar does not support a causal link between sugars consumption and obesity and associated chronic disease. As an example, while sugar consumption decreased in UK, Australia and Canada, the obesity rate grew in their respective populations.

Similarly, the International Food and Beverage Alliance (IFBA)^83^ claimed, without providing evidence in support, that

[…] the UK salt reduction initiative, a public-private partnership led by the UK government which has resulted in the reduction of average daily salt intakes by 15% since 2001. Similar salt reduction initiatives and trans fat and calorie reduction strategies around the world have also proven effective.

The 56 claims with identifiable references were made by only five business associations: the International Council of Beverages Associations (ICBA), IFBA, the Grocery Manufacturers Association (now Consumer Brands Association),^84^ Food Industry Asia and the German Federation for Food Law and Food Science (now Food Federation Germany).^85^ The majority of these claims were made by ICBA who participated in three of the four included consultations.

### Quality of evidence

**Figure 2:**
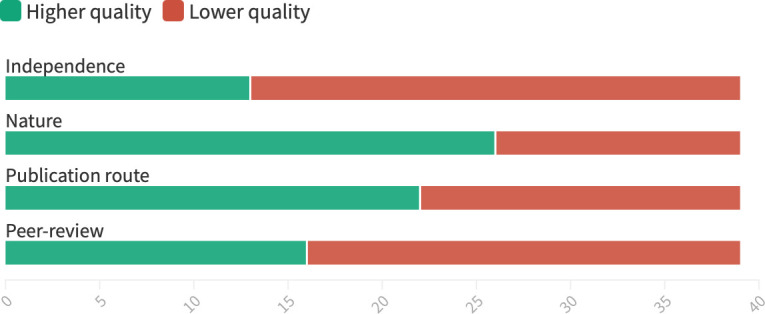
Quality indicators across all 39 pieces of evidence cited to support factual claims. Higher quality was indicated where evidence was clearly independent or appeared independent, was based on research, published in a peer-reviewed journal or by a government/intergovernmental organisation, and was externally peer-reviewed. Created using *flourish studio*.^81^

The 56 claims citing identifiable evidence referred to 39 separate pieces of evidence. Figure 2 summarises overall findings on quality of evidence, showing that although a significant proportion of the evidence cited was research published in higher-quality outlets (academic journals, governmental/international organisations), the majority was neither independent nor peer-reviewed. Only four cited items were independent, peer-reviewed research.

#### Independence

Of these 39 pieces of evidence, just nine (23.1%) were clearly independent, while 13 (33.3%) were industry-funded; 13 (33.3%) were industry-linked, four (10.3%) appeared independent but did not provide sufficient information to conclusively rule out industry links. In most pieces of evidence classified as industry-linked, one or more of the authors had received funding from UPFI entities (see online supplemental file 1 for details). In particular, claims supporting alternatives to regulation (categories 2 and 3)—most often self-regulatory initiatives—heavily relied on evidence that was not independent (75% industry-linked/funded). This encompassed predominantly industry-conducted or commissioned evaluations of their own commitments.

10.1136/bmjgh-2021-006176.supp1 Supplementary data

Of the 13 items of industry-funded or industry-linked evidence which were published in academic journals, only four clearly declared a COI.^86–89^ An additional two reported industry financial contributions, but did so under ‘acknowledgements’ or ‘acknowledgements and disclosures’.^90 91^ Of the rest, four did not have a COI section,^92–95^ while three articles explicitly declared no COI,^96–98^ one thereof not only linked to, but funded by an UPFI entity.^96^

#### Nature of evidence

Of the 39 pieces of evidence, 26 (66.7%) were research, eight (20.5%) were strategy documents, four (10.3%) were opinion pieces, and one (2.6%) was raw data. Notably, 19 of the 26 research-based sources were industry-funded or -linked.

#### Publication route

Of the 39 pieces of evidence, 18 (46.2%) were published by academic outlets, closely followed by private companies and organisations which had published 17 (43.6%). This included reports published by the submitting business associations themselves and evidence from think tanks and research firms such as Oxford Economics^99 100^ and McKinsey Global Institute.^101^ ‘Overcoming obesity: An initial economic analysis’ by the McKinsey Global Institute was also the most referenced piece of evidence across all submissions, cited nine times across five consultation responses by three different business associations. A further four items (10.3%) were published by intergovernmental organisations or governments. Only 16 (41%) pieces of evidence cited to support factual claims were externally peer-reviewed. This is less than the number of items published in peer-reviewed journals, as two referenced conference abstracts do not appear to have undergone external peer review.^89 92^

### Use of scientific evidence

In this section, we address *how* scientific evidence was used to support the factual claims around policy effects. We discuss examples under each core category of claims: questioning regulatory policies (claim categories 1, 1.1, and 2) and promoting alternatives to regulation (claim categories 3 and 3.1).

#### Questioning regulatory policies

Factual claims that regulatory policies do not work or will have negative consequences, although made in 12 responses from seven organisations, were only backed by peer-reviewed research evidence in three responses, all made by one organisation, ICBA. They cited three independent research articles to support five claims that SSB taxation does not work or will have negative consequences,^102–104^ and a fourth to question the link between SSBs and obesity.^105^

To question the effectiveness of the policy, they cited a review by independent scientists, Bes-Rastrollo *et al*,^102^ in their submission to the consultation on *Appendix 3 to WHO Global NCD Action Plan 2013–2020*,^106^ stating that a

recent review summarizing all data related to taxation of sugars found that taxation did not affect obesity rates [reference: Bes-Rastrollo *et al*]. In this summary, it was found that of the six published studies to date where data had been measured (as opposed to modeled), five found no effect of taxation whatsoever, while the sixth found less than a 1.8 kg difference in body weight after 20 years.

This appears to reflect a technique Ulucanlar *et al*^58^ term *misquoting* of evidence, as the original source concludes that the ‘best available scientific evidence suggests that added sugars, especially SSB consumption, are an important risk factor for weight gain and obesity’ and that the ‘tax tool alone on added sugars appears insufficient to curb the obesity epidemic, but it needs to be included in a multicomponent and comprehensive structural strategy to combat obesity’.^102^ While the second part of ICBA’s statement, focusing on observation studies, is accurate, it omits important contextual information, first that most tax rates in the observational sample were lower than the recommended 20% threshold, as remarked by Bes-Rastrollo *et al* themselves, and second, that overall, ‘results found a significant inverse association between SSB excise taxes and weight gain or obesity, although the magnitude of the estimates of effect was small’.^102^

In their submission to the *WHO Independent High-Level Commission on NCDs* and a similar comment on the *Montevideo Roadmap 2018–2030*, ICBA *selectively quoted* a publication by Silver *et al*^104^ to support an argument that SSB taxation would not only fail to reduce SSB consumption but would also increase the consumption of other unhealthy products:

In Berkeley, California, a tax on SSBs has caused calorie intake to rise rather than decrease. For instance, a recent study of the SSB tax implemented in Berkeley, California, found that while caloric consumption of taxed beverages dropped by a statistically insignificant margin of an average of six calories per day – equivalent to a bite of an apple, caloric consumption of untaxed beverages rose by an average of 32 calories per day, resulting in a net increase of 26 calories per person per day resulting from the tax [reference: Silver et al]. In other words, consumers switched from soft drinks to milkshakes, smoothies and other similarly calorie-dense products – resulting in more calories consumed.

In fact, Silver *et al* concluded that one year after the introduction of the Berkeley SSB tax, ‘prices of SSBs increased in many, but not all, settings, SSB sales declined, and sales of untaxed beverages (especially water) and overall study beverages rose in Berkeley’.^104^ The figure reported by ICBA only refers to the self-reported SSB intake which decreased by 19.8% but was statistically insignificant. The authors reported statistically significant results for an increase of 15.6% in water sales and a 9.6% decrease in SSB sales. Self-reports did indicate an increase in caloric intake of untaxed beverages such as milkshakes and yoghurt smoothies, but ICBA failed to address the authors’ observation that this contrasts with the substitution pattern seen in the ‘point-of-sale data, which showed an increase in water sales and smaller but still significant increases in sales of plain milk and untaxed fruit, vegetable, and tea drinks’.^104^

Discussing an independent observational study by Colchero *et al*^103^ on changes in purchasing after the introduction of an SSB tax in Mexico in their submission to the WHO consultation on *Appendix 3 of the Global NCD Action Plan*,^106^ ICBA omitted important qualifying information to suggest that the study demonstrates the ineffectiveness of the policy:

Although one widely-publicized study indicates that purchases of taxed beverages decreased by an average of six percent in 2014 [reference: Colchero *et al*], it is important to note that the calorie consumption from beverages has declined only slightly – roughly between two and six fewer calories per day in a diet of more than 3000 calories per day in Mexico [reference: FAO, National Institute of Statistics and Geography, and National Association of Soft Drink and Carbonated Water Producers], which is a daily caloric decrease of less than one half of one percent.

Although the 6% average figure is correct, ICBA failed to mention that the decline in consumption had grown progressively, reaching 12% by the end of 2014. Moreover, in a footnote, ICBA also appeared to *mimic scientific critique* by insisting on methodological perfectionism, pointing out ostensible methodological flaws to dismiss Colchero *et al*’s findings:

This study had a number of methodological and other limitations. For example: (1) it was an observational study so causality could not be established; (2) rural populations were ignored and traditional stores […] were likely underrepresented (as were the working poor or very poor) since the study was based on Nielsen panel data covering 53 cities each with 50,000 or more residents; (3) the data was based on purchases and not consumption; and (4) the study was not controlled for other environmental factors (eg, information campaigns that could have had a bigger impact than the actual tax).

It is noteworthy that ICBA directed no such critical assessment towards the favourable, typically lower-quality evidence it cited throughout this submission.

ICBA invoked six articles to question the well-established^107^ link between sugar or SSBs and obesity or negative health outcomes. Five of these were either industry-funded or industry-linked.^86–88 90 91^ The sixth, independent study,^105^ was cited correctly to the extent that ICBA echoed the authors’ finding that the evidence on the relationship between SSB consumption and body mass index is mixed if adjusted for total calorie intake. However, a preceding claim by ICBA that ‘the overall weight of the scientific evidence on sugar and/or sugar-sweetened beverages show [sic] that they do not have a unique effect on body weight beyond their contribution to total calorie intake’ is not supported by the article which states that its conflicting results, while potentially weakening confidence in association strength, do not disprove an association between SSBs and obesity. This also appears to be a misquotation of evidence.^58^

#### Promoting alternatives to regulation

IFBA and the Grocery Manufacturers Association were the only organisations to use peer-reviewed research articles in support of self-regulation or co-regulation. They did so in eight instances, and all articles were either industry-funded or industrylinked.^93–97^

IFBA,^83^ for instance, used two academic publications^95 97^ to support its statement that EPODE (Ensemble Prévenons l’Obésité Des Enfants), a public–private programme partly funded by companies such as Nestlé and The Coca-Cola Company,^108^ ‘has shown encouraging results in preventing childhood obesity in France and Belgium and has reduced the socioeconomic gap in obesity prevalence in France’. The first paper by Van Koperen *et al*^97^—which we classified as industry-linked because there was evidence that two of the authors had accepted UPFI funding in the five years before publication—did not set out to examine the effectiveness of EPODE, but to ‘learn more on the dynamics and key elements of the EPODE program tackling childhood overweight and obesity to support future research and evaluation’ and present a logic model.^97^ Similarly, the second paper,^95^ which was supported by The Coca-Cola Company and whose lead author also contributed to the Van Koperen *et al* article, aimed to ‘provide a detailed description of EPODE methodology, including its broad and overarching approach to strengthening and enriching CBIs [community-based interventions] aimed at preventing childhood obesity’.^95^ The article does, however, suggest that decreases in obesity prevalence in EPODE pilot towns are attributable to the programme, with IFBA repeating a statement made in the article’s abstract that EPODE has ‘shown encouraging results in preventing childhood obesity in France and Belgium and has reduced the socioeconomic gap in obesity prevalence in France’.^95^

## Discussion

By exploring UPFI use of evidence in global health governance for the first time, we add to an emerging body of literature investigating how unhealthy commodity industries use evidence to oppose public health regulation.^57–59 62^ In summary, our work indicates that the factual claims UPFI actors made to oppose the regulation of unhealthy products in consultation with the WHO were largely unsupported by high-quality, independent evidence, and where scientific evidence was used, it was often misrepresented.

It is noteworthy that, despite claims to support EBPM and language which mimics scientific reasoning,^63^ over half of the UPFI submissions we analysed did not refer to any evidence. Even among those which did, a significant proportion of claims opposing dietary public health regulation were not supported with any evidence. Where evidence was cited, the majority was neither peer-reviewed nor independent: of 114 factual claims, only 6 were made based on peer-reviewed *and* independent research, all of which misrepresented the original source to some degree. These six claims were all made by the same organisation, ICBA, to oppose SSB taxation. The group, which represents soft drinks producers, submitted some of the longest consultation responses with the most references to evidence, which goes a long way towards explaining the skew of our sample towards SSB taxation.

Overall, the arguments made by UPFI respondents to oppose statutory regulation do not align with the public health evidence. Claims that regulation to address dietary NCDs does not have the desired effect, predominantly levelled at SSB taxation, contradict independent evidence which supports the potential of taxes to favourably influence dietary behaviours.^109–112^ Although the evidence in favour of SSB taxes has grown substantially since the consultations were held, high-quality publications were available when the majority of consultation submissions were written.^113^ UPFI respondents also questioned the links between their products or specific ingredients—predominantly sugar—and obesity or dietary NCD, despite a substantial body of independent evidence which links added sugar intake,^114^ and SSBs in particular,^115–117^ to obesity and a range of NCDs. Similarly, claims of negative economic consequences were primarily made in the context of SSB taxation. While industry-commissioned evidence does tend to report such impacts,^118^ independent research suggests that SBB taxes have not had negative impacts on employment and the wider economy,^118–121^ or even businesses.^112^ Initial evidence suggests that other regulatory policies which were contested by industry—mandatory labelling and advertising restrictions—also work as intended^122 123^ and do not affect employment.^124^

Independent evidence also indicates that self-regulation is not sufficient to address the issues of obesity and dietary NCDs.^4–12^ Self-regulation of advertising, for instance, does not appear to be effective enough to reduce children’s exposure to unhealthy food adverts^6 8 125–128^ and industry codes have widely been criticised as weak by public health researchers.^6 129^ In line with evidence which suggests that industry-funded research results in more favourable conclusions,^51–55^ industry evaluations of self-regulatory codes tend to report much higher effectiveness and compliance than independent evaluations.^128^ This may explain our observation that the vast majority of claims in favour of self-regulatory or co-regulatory approaches relied on industry-produced or industry-commissioned materials.

When assessing the independence of cited evidence, we found it remarkable that the majority of industry-funded or industry-linked academic articles did not declare a COI. While some simply did not contain a COI section, others explicitly declared that they had no COI. Of those that did declare an interest, this was at times combined with the acknowledgements. This exemplifies why current reporting practices are inadequate and highlights the urgent need for enforced and structured COI reporting processes within and beyond public health.^130^

There are indications that commercial actors draw on a shared set of preferred evidence and consultancies across levels of governance and policy settings. For instance, reports by the research firm Oxford Economics^99 100^—an organisation with a history of producing reports for the tobacco industry^131^—were also cited by respondents to the South African SSB tax consultation.^62^ The most frequently cited item in our study, a discussion paper funded and written by the McKinsey Global Institute^101^ which ranks taxation and media restrictions as low-impact interventions to address obesity, but concludes that only comprehensive measures will work to tackle obesity, has also been cited in other policy debates to oppose public health regulation.^64 132^

In addition to the agnogenic practices described above, casual mentions of ‘the evidence’ or ‘science’ without reference to concrete evidence, as well as vague expressions of alignment with EBPM, appear to form part of attempts to position industry as a legitimate actor in public health policy. Sitting beyond the instrumental role of evidence, this rhetorical facet may play a role in lending discursive power and credibility to policy actors.

Overall, our findings confirm existing research on the use of evidence by unhealthy commodity industries in public health policy,^59–61 133^ adding to a growing body of literature which indicates high levels of coherence in practices across sectors.^134–137^ This warrants reconsideration of engagement with actors who hold a clear commercial interest in a deregulated food system, perhaps towards an approach more coherent with Article 5.3 of the WHO Framework Convention on Tobacco Control which demands that public health policymaking is protected from the vested interests of the tobacco industry.^138^ Where engagement does take place, adjustments to consultation processes could be made, both to encourage the use of higher-quality evidence (and recognition were there is none) and to enable those developing policies or policy recommendations to more readily assess cited sources. One way to achieve this may be to require consultation respondents to declare origins and funding of referenced evidence, particularly where the submitting organisation itself has financially supported the research or researchers. Consultation documents may also ask respondents to provide evidence in support of their claims in a structured way and directly attach sources where these are not publicly available, thus facilitating evidence appraisal by policymakers.

### Limitations

Our results on evidence use were heavily driven by a small set of actors who referenced large amounts of evidence, whereas the majority referenced little or no evidence. This may limit their generalisability of our findings. While we went beyond declarations in the cited sources to identify industry links to the evidence, our web-based investigation is unlikely to have identified all extant connections. Our research focused on UPFI actors and does not compare how non-industry actors such as Member States or civil society used evidence in their submissions. We concentrated on this subset of respondents due to the inherent conflict between the interests of the UPFI and public health, which has manifested in UPFI opposition to policies needed to address the considerable burden of obesity and NCDs.^34 63 139^

## Conclusions

Our findings suggest that UPFI actors’ rhetorical alignment with EBPM^63^ remains mere rhetoric in a majority of cases. Stakeholder consultation, while potentially valuable in that it allows communities and civil society to feed into policy documents, also explicitly gives a voice to the often better-resourced industries whose products are at threat of being regulated. This becomes an issue when—as shown in this study—industry actors question the benefits and emphasise the costs of public health regulation while supporting their preferred alternatives, largely by promoting low-quality evidence or misrepresenting higher-quality evidence. Thus, it is important to critically evaluate the claims made and evidence used in consultation submissions, a process which is time-consuming and would pose a substantial burden on policymakers. On a practical level, this might be eased through clear reporting requirements and thresholds regarding the quality and independence of evidence. This does not, however, address the less tangible but potentially powerful gain of legitimacy which commercial actors may achieve by aligning themselves with the ideal of EBPM. In light of similar conduct of other unhealthy commodity industries, it is worth questioning the value engagement with commercial interests adds to policy development. This is particularly pertinent as resources could instead be invested into redressing power asymmetries in global health governance, for instance, by more actively involving less politically powerful parts of the food system.

## Data Availability

Data are available in a public, open access repository. All data analysed for this study are in the public domain.
