# Supplementary material for: Commercial use of evidence in public health policy: a critical assessment of food industry submissions to global-level consultations on non-communicable disease prevention
Source: BMJ Glob Health. 2021 Aug 22;6(8):e006176. doi: 10.1136/bmjgh-2021-006176 (PMC8383892; doi:10.1136/bmjgh-2021-006176)
Supplement: Supplementary data [file bmjgh-2021-006176supp001.pdf]

Supplementary File 1: All pieces of evidence cited to support core claims around regulation.

Consultations: Appendix 3 = *Updating Appendix 3 of the WHO Global NCD Action Plan 2013–2020*; Shanghai = *Zero draft Shanghai Declaration on Health Promotion*; Montevideo = *Consultation on the Member State-led draft outcome document for WHO Global Conference on NCDs (Montevideo Roadmap)*; HLC = *Web-based consultation of the WHO Independent High-level Commission on NCDs*.
Claims: R1 = *regulation does not work*; R2 = *regulation will have negative consequences*; R3 = *rationale for regulation is flawed*; AR = *alternatives to regulation work (better)*; AR-C = *compliance with alternatives to regulation is high*.

Nr.	Title	Citing actor(s): consultation	Claim(s) made	Type of evidence	Publication route	Independence	Externally peer-reviewed?
1	Accenture (2009-2011). Compliance Monitoring Reports for the International Council of Beverages Associations on Global Advertising in Television, Print and Internet. Washington, DC: ICBA.	ICBA: Appendix 3	AR-C	Strategy document	Publication by private companies and organisations	Industry-funded: Commissioned by ICBA	Not peer-reviewed
2	Accenture (2012). *Compliance Monitoring Report for the International Council of Beverages Associations on Global Advertising in Television, Print and Internet.* Washington, DC: ICBA.	ICBA: Appendix 3	AR-C	Strategy document	Publication by private companies and organisations	Industry-funded: Commissioned by ICBA	Not peer-reviewed
3	Accenture (2016*). 2015 Compliance Monitoring Report for the International Food & Beverage Alliance on Global Advertising in Television, Print and Internet*. Geneva: IFBA.	FIA: Shanghai	AR-C	Strategy document	Publication by private companies and organisations	Industry-funded: Commissioned by IFBA	Not peer-reviewed
4	Aguilar, A., Gutierrez, E., & Seira, E. (2015). *Taxing calories in Mexico: preliminary and incomplete draft*. Mexico City: Center for Economic Research, Autonomous Technological Institute of Mexico.	ICBA: Appendix 3	R1	Research	Publication by private companies and organisations	Industry-linked: The paper originated from a collaboration with the food industry group ConMéxico	Not peer-reviewed
5	Bes-Rastrollo, M., Sayon-Orea, C., Ruiz-Canela, M., & Martinez-Gonzalez, M. A. (2016). Impact of sugars and sugar taxation on body weight control: A comprehensive literature review. *Obesity*, 24(7), 1410-1426. DOI: 10.1002/oby.21535	ICBA: Appendix 3	R1	Research	Peer-reviewed journals and other academic outlets	Clearly independent	Peer-reviewed
6	Borys, J. M., Valdeyron, L., Levy, E., Vinck, J., Edell, D., Walter, L., ... & Barriguette, A. (2013). EPODE–a model for reducing the incidence of obesity and weight-related comorbidities. *European endocrinology*, 9(2), 116. DOI: 10.17925/EE.2013.09.02.116	IFBA: Shanghai	AR	Research	Peer-reviewed journals and other academic outlets	Industry-funded: Article supported by The Coca-Cola Company	Peer-reviewed
7	British Soft Drinks Association (2016). *Sugar intake from soft drinks is falling year on year*. London: British Soft Drinks Association.	ICBA: Appendix 3	R1	Opinion	Publication by private companies and organisations	Industry-funded: Authored and released by British Soft Drinks Association	Not peer-reviewed
8	Canadian Beverage Association (2016). *Canadian Beverage Association statement in response to calls for taxation on sugar-sweetened and artificially-sweetened beverages*. Sarasota, FL: Newswire.	ICBA: Appendix 3	R1	Opinion	Publication by private companies and organisations	Industry-funded: Authored and released by Canadian Beverage Association	Not peer-reviewed
9	Colchero, M. A., Popkin, B. M., Rivera, J. A., & Ng, S. W. (2016). Beverage purchases from stores in Mexico under the excise tax on sugar sweetened beverages: observational study. *BMJ, 352.* DOI: 10.1136/bmj.h6704	ICBA: Appendix 3	R1	Research	Peer-reviewed journals and other academic outlets	Clearly independent	Peer-reviewed
10	Commission on Ending Childhood Obesity (2016). *Report of the Commission on Ending Childhood Obesity*. Geneva: World Health Organization.	GMA	R1	Strategy document	Official IGO or government publications	Clearly independent	Not peer-reviewed

		(now Consumer Brands Association): Appendix 3					
**11**	Dietary Guidelines Advisory Committee (2015). *Scientific Report of the 2015 Dietary Guidelines Advisory Committee*. Washington, DC: U.S. Department of Agriculture.	ICBA: Appendix 3	R3	Strategy document	Official IGO or government publications	Clearly independent	Not peer-reviewed
**12**	Dobbs, R., Sawers, C., Thompson, F., Manyika, J., Woetzel, J. R., Child, P., ... & Spatharou, A. (2014). *Overcoming obesity: an initial economic analysis.* New York, NY: McKinsey Global Institute.	IFBA: Montevideo & Shanghai; FIA: Montevideo; ICBA: HLC & Montevideo	AR (x3), R1 (x2)	Research	Publication by private companies and organisations	Appears independent	Not peer-reviewed
**13**	Drenkard, S. & Shupert, C. (2017). Soda Tax Experiment Failing in Philadelphia Amid Consumer Angst and Revenue Shortfalls. *Fiscal Fact, 555.*	ICBA: Montevideo & HLC	R2 (x2)	Research	Publication by private companies and organisations	Industry-linked: Publishing Tax Foundation board of Directors included PepsiCo executive at time of writing.	Not peer-reviewed
**14**	Economist Intelligence Unit (2017). *Tackling obesity in ASEAN: Prevalence, impact, and guidance on interventions*. London: Economist Intelligence Unit.	FIA: Montevideo	AR	Research	Publication by private companies and organisations	Industry-linked: Commissioned by ARoFIIN, a partnership FIA is a member of	Not peer-reviewed
**15**	Ecorys (2014). *Food taxes and their impact on competitiveness in the agri-food sector, a study*. Brussels: European Competitiveness and Sustainable Industrial Policy Consortium.	ICBA: Appendix 3; BLL: Montevideo	R1 & R2 (x2)	Research	Publication by private companies and organisations	Appears independent	Not peer-reviewed
**16**	Fairhead, H. (2016). *The unintended consequences of the sugar tax*. London: TaxPayers' Alliance.	ICBA: HLC	R2	Opinion	Publication by private companies and organisations	Appears independent (intransparent funding)	Not peer-reviewed
**17**	Food Industry Asia (2016). *Fast Facts on Packs: GDA Nutrition Labelling Report 2015*. Singapore: Food Industry Asia.	FIA: Montevideo, Shanghai, & HLC	AR-C (x3)	Strategy document	Publication by private companies and organisations	Industry-funded: Commissioned by FIA	Not peer-reviewed
**18**	Gadah, N. S., Kyle, L. A., Smith, J. E., Brunstrom, J. M., & Rogers, P. J. (2016). No difference in compensation for sugar in a drink versus sugar in semi-solid and solid foods. *Physiology & behavior*, 156, 35-42. DOI: 10.1016/j.physbeh.2015.12.025	ICBA: Appendix 3	R3	Research	Peer-reviewed journals and other academic outlets	Industry-funded: The research was funded by Sugar Nutrition UK (now disbanded). The authors thank British Sugar employees for study input; last author declares Coca-Cola and International Sweeteners Association funding.	Peer-reviewed

**19**	Ha, V., Cozma, A. I., Choo, V. L., Mejia, S. B., de Souza, R. J., & Sievenpiper, J. L. (2015). Do fructose-containing sugars lead to adverse health consequences? Results of recent systematic reviews and meta-analyses. *Advances in Nutrition*, 6(4), 504S-511S. DOI: 10.3945/an.114.007468	ICBA: Appendix 3	R3	Research	Peer-reviewed journals and other academic outlets	Industry-linked: Multiple authors declare funding from food industry entities in the 'author disclosures' section.	Peer-reviewed
**20**	Hanks, A., Wansink, B., Just, D., Smith, L., Cawley, J., Kaiser, H., ... & Schulze, W. (2013). From Coke to Coors: a field study of a fat tax and its unintended consequences. Journal of Nutrition Education and Behavior, 45(4), S40. DOI: 10.1016/j.jneb.2013.04.108	ICBA: Appendix 3	R1, R2	Research (conference abstract)	Peer-reviewed journals and other academic outlets	Industry-linked: 1st & 2nd author declared McDonald's funding in the same year (see e.g., here).	Not peer reviewed
**21**	Kahn, R., & Sievenpiper, J. L. (2014). Dietary sugar and body weight: have we reached a crisis in the epidemic of obesity and diabetes?: we have, but the pox on sugar is overwrought and overworked. *Diabetes Care*, 37(4), 957-962. DOI: 10.2337/dc13-2506	ICBA: Appendix 3	R3	Research	Peer-reviewed journals and other academic outlets	Industry-funded: Funding declaration includes grants from The Coca-Cola Company and other food industry entities	Peer-reviewed
**22**	Kees, J., & Fitzgerald, M. P. (2016). Who Uses Facts Up Front? A Baseline Examination of Who is Using Standardized Front-of-Package Nutrition Disclosures. *Journal of Consumer Affairs, 50*(2), 458-470. DOI: 10.1111/joca.12090	GMA: Appendix 3 & Montevideo	AR (x2)	Research	Peer-reviewed journals and other academic outlets	Industry-linked: 1st author was working as a consultant for the Grocery Manufacturers Association at the time	Peer-reviewed
**23**	Kees, J., Royne, M. B., & Cho, Y. N. (2014). Regulating front-of-package nutrition information disclosures: A test of industry self-regulation vs. other popular options. *Journal of Consumer Affairs, 48*(1), 147-174. DOI: 10.1111/joca.12033	GMA: Appendix 3 & Montevideo	AR (x2)	Research	Peer-reviewed journals and other academic outlets	Industry-linked: 1st author was working as a consultant for the Grocery Manufacturers Association at the time	Peer-reviewed
**24**	Kolish, D., Enright, M., & Oberdorff, B. (2014). The Children's Food and Beverage Advertising Initiative in Action: A Report on Compliance and Progress During 2013. Arlington, VA: Council of Better Business Bureaus.	GMA: Appendix 3 & Montevideo	AR-C (x2)	Strategy document	Publication by private companies and organisations	Industry-funded: No clear note on funding but published by CFBAI and written by its staff. CFBAI is a self-regulatory programme by food and beverage producers.	Not peer-reviewed
**25**	Mexico's National Health Survey (2016), ENSANUT	ICBA: Montevideo & HLC	R1	Data without analysis	Official IGO or government publications	Clearly independent	Not peer-reviewed
**26**	Noronha, J. C., Choo, V., Mejia, S. B., Viguiliouk, E., Jayalath, V., Braunstein, C., ... & Sievenpiper, J. (2016). Liquid Calories from Sugars Do Not Increase Body Weight More than Solid Calories: A Systematic Review and Meta-Analysis of Controlled Feeding Trials. The FASEB Journal, 30(1_supplement), 906-6.	ICBA: Appendix 3	R3	Research (conference abstract)	Peer-reviewed journals and other academic outlets	Industry-linked: Toronto 3D Knowledge Synthesis and Clinical Trials Foundation (listed as affiliation for all authors) received multiple Coca-Cola grants between 2014-2016; Sievenpiper has	Not peer-reviewed

						received Coca-Cola funding; Kendall 2declared exhaustive conflicts of interest	
**27**	Oxford Economics (2017). The economic impact of philadelphia's beverage tax. https://www.oxfordeconomics.com/my-oxford/projects/426008	ICBA: HLC	R2	Research	Publication by private companies and organisations	Industry-funded	Not peer-reviewed
**28**	Oxford Economics & International Tax and Investment Center (2016) *The impact of selective food and non-alcoholic beverage taxes*. Oxford: Oxford Economics.	ICBA: Appendix 3 & HLC	R1 (x2), R2	Research	Publication by private companies and organisations	Industry-linked: ITIC founding members include PepsiCo; OE clients include The Coca-Cola Company & PepsiCo	Not peer-reviewed
**29**	Quirmbach, D., Cornelsen, L., Jebb, S. A., Marteau, T., & Smith, R. (2018). Effect of increasing the price of sugar-sweetened beverages on alcoholic beverage purchases: an economic analysis of sales data. *J Epidemiol Community Health, 72*(4), 324-330.	ICBA: HLC	R2	Research	Peer-reviewed journals and other academic outlets	Industry-linked: 3rd author has received industry funding for a number of projects between 2004 & 2015.	Peer-reviewed
**30**	Rippe, J. M., & Angelopoulos, T. J. (2016). Added sugars and risk factors for obesity, diabetes and heart disease. *International Journal of Obesity*, 40(S1), S22. DOI: 10.1038/ijo.2016.10	ICBA: Appendix 3	R3	Research	Peer-reviewed journals and other academic outlets	Industry-linked: 1st author declares funding from food industry entities including Kraft Foods, PepsiCo, and Coca-Cola under *competing interests*.	Peer-reviewed
**31**	Sarlio-Lähteenkorva, S., & Winkler, J. T. (2015). Could a sugar tax help combat obesity?. *BMJ*, 351, h4047. DOI: 10.1136/bmj.h4047	ICBA: Appendix 3	R1, R2	Opinion	Peer-reviewed journals and other academic outlets	Clearly independent	Peer-reviewed
**32**	Silver, L. D., Ng, S. W., Ryan-Ibarra, S., Taillie, L. S., Induni, M., Miles, D. R., ... & Popkin, B. M. (2017). Changes in prices, sales, consumer spending, and beverage consumption one year after a tax on sugar-sweetened beverages in Berkeley, California, US: A before-and-after study. *PLoS medicine, 14*(4), e1002283. DOI: 10.1371/journal.pmed.1002283	ICBA: Montevideo & HLC	R1 (x2), R2	Research	Peer-reviewed journals and other academic outlets	Clearly independent (note: last author was co-investigator on a Nestle Waters funded project >5 years previously)	Peer-reviewed
**33**	Smith Edge, M., Toner, C., Kapsak, W. R., & Geiger, C. J. (2014). The impact of variations in a fact-based front-of-package nutrition labeling system on consumer comprehension. *Journal of the Academy of Nutrition and Dietetics*, *114*(6), 843. DOI: 10.1016/j.jand.2014.01.018	GMA: Montevideo & Appendix 3	AR (x2)	Research	Peer-reviewed journals and other academic outlets	Industry-funded: Funded by the Grocery Manufacturers of America with a grant to the International Food Information Council Foundation	Peer-reviewed
**34**	Soon, G., Koh, Y. H., Wong, M. L., & Lam, P. W. (2008). *Obesity Prevention and Control Efforts in Singapore*. Singapore: The National Bureau of Asian Research.	FIA: Shanghai	AR	Research	Publication by private companies and organisations	Industry-linked: NBR's funders include Starbucks	Not peer-reviewed
**35**	Trumbo, P. R., & Rivers, C. R. (2014). Systematic review of the evidence for an association between sugar-sweetened beverage consumption and risk of obesity. Nutrition Reviews, 72(9), 566-574. DOI: 10.1111/nure.12128	ICBA: Appendix 3	R3	Research	Peer-reviewed journals and other academic outlets	Clearly independent (note: the journal is published by the	Peer-reviewed

						International Life Sciences Institute)	
**36**	Van Koperen, T. M., Jebb, S. A., Summerbell, C. D., Visscher, T. L. S., Romon, M., Borys, J. M., & Seidell, J. C. (2013). Characterizing the EPODE logic model: unravelling the past and informing the future. Obesity reviews, 14(2), 162-170. DOI: 10.1111/j.1467-789X.2012.01057.x	IFBA: Shanghai	AR	Research	Peer-reviewed journals and other academic outlets	Industry-linked: 2nd author has received industry funding for a number of projects between 2004 & 2015. 6th author published Coca-Cola funded work in the same year.	Peer-reviewed
**37**	World Health Organization (2017). *Tackling NCDs: 'Best buys' and other recommended interventions for the prevention and control of noncommunicable diseases*. Geneva: World Health Organization.	ICBA: Montevideo & HLC	R1 (x2)	Strategy document	Official IGO or government publications	Clearly independent	Not peer-reviewed
**38**	Wilson, P. & Hogan, S. (2017). *Sugar taxes: a review of the evidence. NZIER report to Ministry of Health*. Wellington: NZ Institute of Economic Research.	ICBA: HLC	R1	Research	Publication by private companies and organisations	Appears independent	Not peer-reviewed
**39**	Wittekind, A., & Walton, J. (2014). Worldwide trends in dietary sugars intake. Nutrition research reviews, 27(2), 330-345. DOI: 10.1017/S0954422414000237	ICBA: Appendix 3	R3	Research	Peer-reviewed journals and other academic outlets	Industry-funded: Supported by the World Sugar Research Organisation, an industry-funded body	Peer-reviewed
